# A new picture of cell wall protein dynamics in elongating cells of *Arabidopsis thaliana*: Confirmed actors and newcomers

**DOI:** 10.1186/1471-2229-8-94

**Published:** 2008-09-16

**Authors:** Muhammad Irshad, Hervé Canut, Gisèle Borderies, Rafael Pont-Lezica, Elisabeth Jamet

**Affiliations:** 1 Surfaces cellulaires et Signalisation chez les Végétaux, UMR 5546 CNRS – UPS-Université de Toulouse, Pôle de Biotechnologie Végétale, 24 chemin de Borde-Rouge, BP 42617 Auzeville, 31326 Castanet-Tolosan, France

## Abstract

**Background:**

Cell elongation in plants requires addition and re-arrangements of cell wall components. Even if some protein families have been shown to play roles in these events, a global picture of proteins present in cell walls of elongating cells is still missing. A proteomic study was performed on etiolated hypocotyls of *Arabidopsis *used as model of cells undergoing elongation followed by growth arrest within a short time.

**Results:**

Two developmental stages (active growth and after growth arrest) were compared. A new strategy consisting of high performance cation exchange chromatography and mono-dimensional electrophoresis was established for separation of cell wall proteins. This work allowed identification of 137 predicted secreted proteins, among which 51 had not been identified previously. Apart from expected proteins known to be involved in cell wall extension such as xyloglucan endotransglucosylase-hydrolases, expansins, polygalacturonases, pectin methylesterases and peroxidases, new proteins were identified such as proteases, proteins related to lipid metabolism and proteins of unknown function.

**Conclusion:**

This work highlights the CWP dynamics that takes place between the two developmental stages. The presence of proteins known to be related to cell wall extension after growth arrest showed that these proteins may play other roles in cell walls. Finally, putative regulatory mechanisms of protein biological activity are discussed from this global view of cell wall proteins.

## Background

Plant cell walls are dynamic compartments whose composition and structure vary during development or in response to environmental stresses. Variability has been observed in developing roots at the level of glycoproteins in carrot [1], and of polysaccharides in *Arabidopsis thaliana *[2]. In both articles, antibodies against specific epitopes showed an irregular distribution among cell types, as well as changes in the course of development. Cell wall plasticity is particularly needed during cell elongation since cell walls need to expand. During the elongation process, the cellular machinery has to synthesize and export cell wall components and reorganize cell wall networks [3].

To gain information on the genes and proteins involved in cell wall elongation, it is important to dissect the different phases of the process. *Arabidopsis *hypocotyls are a suitable material, since almost no cell division occurs and only the cells present in the embryo undergo elongation [4-6]. Another advantage is that in etiolated hypocotyls, synthesis, addition, and reorganization of cell wall material occur in time-separate phases [4,7]. During the first 3 days after germination synthesis and deposition of cell wall material which result in cell wall thickening, are the main features. Through the following days, the hypocotyl will grow, mainly through extensive cell wall polymer disassembly and rearrangement.

Understanding the molecular mechanisms responsible for rearrangements of cell wall polymers during hypocotyl growth requires the identification of the proteins present *in muro*. On the one hand, previous studies identified gene families involved in rearrangements of cell wall components during cell elongation [8-11]. On the other hand plant cell wall proteomics emerged a few years ago, providing information on cell wall proteins (CWPs) present in different types of *Arabidopsis *cells including cell suspension cultures, roots, rosette leaves, and stems [12,13]. In most cases, limitations were encountered, *e.g*. the presence of intracellular contaminants [14] and a poor quality of separation of CWPs by two-dimensional electrophoresis (2D-E) [15]. The main families of CWPs were identified [16] on the basis on bioinformatics prediction of functional domains.

In this paper, we analyzed the cell wall proteome of half- and fully-grown etiolated hypocotyls, corresponding to the phases of active elongation and after growth arrest. A new strategy was established for CWP separation, and semi-quantification. The comparison of these two proteomes revealed significant dynamics in CWPs. The expected presence of CWPs involved in polysaccharide rearrangement and modification was confirmed in growing hypocotyls. However, some of CWPs were also present in fully-grown hypocotyls, suggesting that either they have long half-lives, or that they could be involved in other functions. Finally, this study led to the identification of new candidates acting in cell elongation.

## Methods

### Plant material

*Arabidopsis thaliana *(ecotype Columbia 0) seeds are purchased from The Nottingham Arabidopsis Stock Centre (NASC) . One hundred and thirty mg of *Arabidopsis *seeds were cultivated in Magenta boxes as previously described [14]. Magenta boxes were kept in the dark at 4°C during 48 h, and subsequently exposed to light for 4 h to synchronize germination. Finally, seedlings were grown in the dark at 23°C for 5 or 11 days. Seedling hypocotyls were cut just below the cotyledons and above the roots. Typically, 36 and 18 Magenta boxes were required for 5 and 11 day-old seedlings respectively.

### Preparation of a cell wall fraction and protein extraction

Cell walls were prepared as previously described [14]. Proteins were extracted from the cell wall fraction in two successive steps, first with a CaCl_2 _solution (5 mM sodium acetate buffer, pH 4.6, 0.2 M CaCl_2 _and 10 μL protease inhibitor cocktail, Sigma), followed by two extractions with a LiCl solution (5 mM sodium acetate buffer, pH 4.6, 2 M LiCl and 10 μL protease inhibitor cocktail). Finally, proteins were desalted and lyophilized.

### Protein separation by cation exchange chromatography

Lyophilized proteins were dissolved in a total volume of 2 mL of water. They were quantified with the Coomassie^® ^protein assay reagent kit (Pierce) using bovine serum albumin (BSA) as a standard [17]. One mg of proteins was used for chromatographic fractionation on a 1 mL HiTrap™ SP FF (Sepharose Fast Flow) column (Amersham Biosciences), equilibrated with 50 mM MES (pH 5.6) operated with an FPLC™ (Fast Protein Liquid Chromatography) System (Amersham Biosciences), controlled by the FPLCdirector™ version 1.0 (Amersham Biosciences). The protein solution was adjusted to 50 mM MES (pH 5.6), and 20 μL protease inhibitor cocktail (Sigma) were added before loading onto the column at a flow rate of 0.5 mL.min^-1^. A 10 mL unfixed fraction was collected at the same rate. Three mL of first wash with 50 mM MES (pH 5.6) were collected at a flow rate of 1 mL.min^-1^. Fixed proteins were eluted by a gradient from 0 M to 0.8 M NaCl in 50 mM MES (pH 5.6); 24 fractions (1 mL each) were collected at a flow rate of 1 mL.min^-1^. Finally the column was successively washed with 3 mL of 1.2 M NaCl and 3 mL of 1.5 M NaCl in 50 mM MES (pH 5.6) at the same flow rate. These washes were also collected in 6 fractions (1 mL per tube). To prevent protein degradation, 2 μL of protease inhibitor cocktail (Sigma) were added to all the 1 mL fractions, 6 μL to the first wash and 20 μL to the unfixed fractions. The fractions were combined in twos and threes, depending on their protein concentration. Non-fixed proteins were concentrated by successive centrifugations at 4000 × *g *using the Centriprep^® ^centrifugal filter device (YM-10 kDa membrane for volumes greater than 6 mL or 5 kDa for smaller volumes, Millipore) at 4000 × *g*. All protein fractions were desalted prior to lyophilization using Econo-Pac^® ^10DG columns (Bio-Rad) equilibrated with 0.2 M ammonium formate.

### Protein separation by mono-dimensional electrophoresis (1D-E) and identification

Each lyophilized fraction was dissolved in 200 μL water and separated by electrophoresis according to Laemmli [18]. Samples were loaded on 22 × 15 × 0.15 cm SDS-polyacrylamide gel with a concentration of 12.5% of acrylamide. The staining was carried out with a Coomassie Brilliant Blue (CBB)-based method [19]. Colored bands were excised from gels and digested with trypsin as described before [20,21]. Matrix-assisted laser desorption ionization – time of flight (MALDI-TOF) mass spectrometry (MS) analyses were performed as previously reported [20,21]. MALDI-TOF-TOF MS analysis was performed using a MALDI TOF-TOF Voyager 4700 (AppliedBiosystems/MDS Sciex, USA). N-terminal sequencing of the protein encoded by *At5g14920 *was performed at *Plate-Forme d'Analyse et de Microséquençage des Protéines *at the Institut Pasteur (Paris, France).

### Semi-quantification

Peptide mass fingerprints were compared to the non-redundant database of *Arabidopsis *of NCBI  using ProteinProspector (MS-FIT: ). A quantification index (QI) was calculated for each protein by adding the percentages of coverage, using peptide mass mapping (ratio between the number of amino acids in peptides detected by MS and the total number of amino acids of the protein) in all the bands of the FPLC profile in which the protein had been identified.

### Bioinformatic analyses

Sub-cellular localization, length of signal peptides, prediction of transmembrane domains, homologies to other proteins and protein functional domains were predicted as described before [15]. Glycoside hydrolases (GHs) and carbohydrate esterases (CEs) were respectively classified according to the CAZy database [22]. Xyloglucan endotransglucosylase-hydrolases (XTHs) and expansins were respectively named according to  and . Arabinogalactan proteins (AGPs) and fasciclin arabinogalactan proteins (FLAs) were named according to Schultz *et al*. [23], Johnson *et al*. [24] and van Engels and Roberts [25]. Proteins homologous to COBRA, leucine-rich repeat extensins (LRXs) and Hyp/Pro-rich proteins were respectively annotated according to Roudier *et al*. [26], Baumberger *et al*. [27], and Fowler *et al*. [28]. Peroxidases were named according to the PeroxiBase [29]. Laccases were annotated as in Pourcel *et al*. [30] and McCaig *et al*. [31]. SKU-like proteins and phytocyanins were respectively named according to Jacobs and Roe [32], and Nersissian and Shipp [33]. Proteases were annotated according to the MEROPS database .

## Results

### Establishment of methods for efficient proteomic analysis of hypocotyl cell walls

The aim of this proteomic study was to compare two developmental stages of *Arabidopsis *hypocotyls. This goal required about 40 g of homogenous material obtained in the following culture conditions: synchronized germination, high seedling density on the culture medium, and control of *in vitro *culture conditions. Five day-old elongating hypocotyls and 11 day-old fully-grown hypocotyls were compared [4,7]. First, the cell wall fraction was prepared to minimize intracellular contamination and loss of proteins [14]. To collect mg amounts of proteins for FPLC separation, two successive extractions were performed with 0.2 M CaCl_2 _and 2 M LiCl [14]. Both extracts were combined and used for further analysis. Typically, about 1 mg of proteins was obtained from 1 g of dry cell wall fraction.

**Figure 1:**
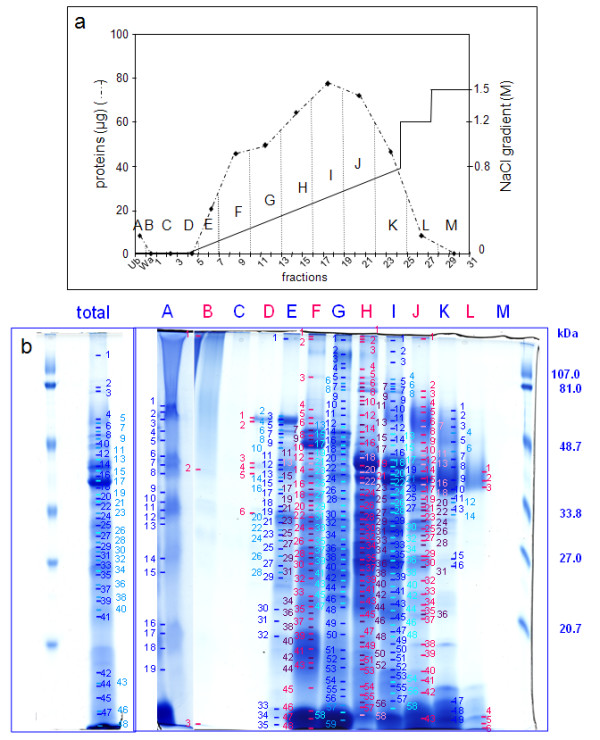
**Analysis of proteins extracted by CaCl_2 _and LiCl from the cell wall fraction prepared from 5 day-old hypocotyls. a**. Separation of proteins by cation exchange chromatography. The graph represents amounts of proteins in each fraction eluted by a NaCl gradient (from 0 M to 0.8 M), followed by two steps at 1.2 M and 1.5 M NaCl. Dotted vertical lines show the grouping of chromatography fractions. Ub stands for unfixed fraction, Wa for washes of the column prior to protein elution, numbering to FPLC fractions, and letters (from A to M) to pools analyzed by 1D-E. **b**. Separation by 1D-E of the total protein extract (total) and of fractions A to M obtained after cation exchange chromatography. Molecular mass markers are on the right. Numbers refer to bands analyzed by MALDI-TOF MS with successful identification (see Additional data file 1).

**Figure 2:**
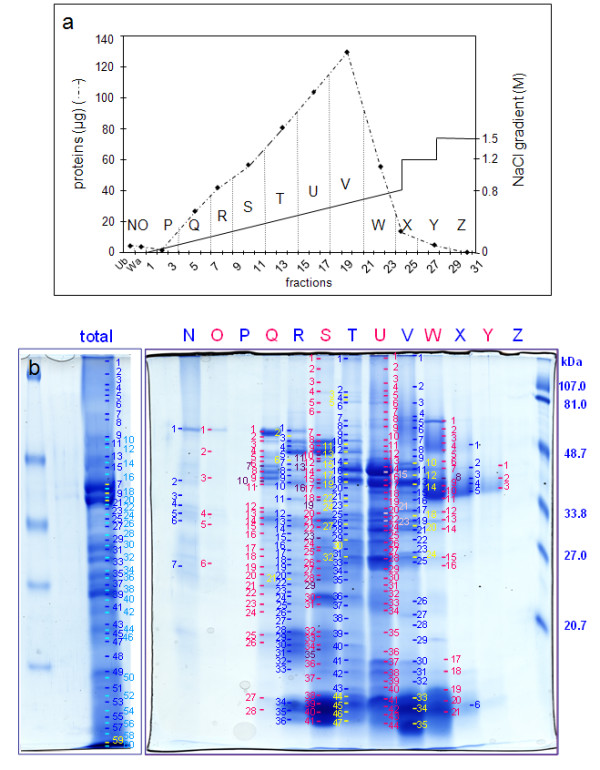
**Analysis of proteins extracted by CaCl_2 _and LiCl from the cell wall fraction prepared from 11 day-old hypocotyls.a**. Separation of proteins by cation exchange chromatography. The graph shows amounts of proteins in each fraction eluted by a NaCl gradient (from 0 M to 0.8 M) followed by two steps at 1.2 M and 1.5 M NaCl. Dotted vertical lines show the grouping of chromatography fractions. Ub stands for unfixed fraction, Wa for washes of the column prior to protein elution, numbering to FPLC fractions, and letters (from N to Z) to pools analyzed by 1D-E. **b**. Separation by 1D-E of the total protein extract (total) and of fractions N to Z obtained after cation exchange chromatography. Molecular mass markers are on the right. Numbers refer to bands analyzed by MALDI-TOF MS with successful identification (see Additional data file 2).

Finally, proteins were separated prior to their identification by peptide mass mapping using MALDI-TOF MS and bioinformatics. Since 2D-E is not appropriate for resolving CWPs [12], these proteins were separated using mono-dimensional gel electrophoresis (1D-E). Approximately 60 bands were stained with Coomassie Brilliant Blue (CBB). Fifty two and 67 proteins were respectively identified in the extract from 5 and 11 day-old hypocotyls extracts (Additional data files 1, 2). Because of the limited resolution by 1D-E, many proteins, some of which have a low number of peptides, were identified in each band. In order to improve the separation and identification of proteins, we introduced an additional step prior to 1D-E. Since most CWPs are basic [16], a cation exchange chromatography was performed using an FPLC device (Figures 1A, 2A). Fractions were collected and combined prior to separation by 1D-E (Figures 1B, 2B). At this point, approximately 500 bands were stained with CBB and were further analyzed by MALDI-TOF MS. The proportion of successful protein identification in stained bands was about 70%. Respectively 141 and 109 proteins were identified in 5 and 11 day-old hypocotyls. Many of the proteins were identified in several bands, thus reinforcing their identification. There was a great improvement in the quality of the analysis: (i) the number of identified proteins was doubled; (ii) the quality of the identifications was improved with higher numbers of peptides for identification of most proteins (Additional data files 3 and 4); (iii) the semi-quantification of proteins allowed the comparison of the two samples.

### Proteins identified in cell wall extracts of Arabidopsis etiolated hypocotyls

Combining results of 1D- and 2D-separation, 147 and 126 proteins were identified respectively in 5 and 11 day-old etiolated hypocotyls respectively (Table 1, Additional data file 5). On the one hand, bioinformatics prediction indicated that 120 (82%) and 101 (79%) proteins were secreted proteins in 5 and 11 day-old hypocotyls respectively. On the other hand, 27 and 25 proteins (in 5 and 11 day-old hypocotyls respectively) had no predicted signal peptide, and were considered as intracellular contaminants. Altogether, 173 proteins were identified in hypocotyls among which 137 (79%) were predicted to be secreted, indicating the good quality of cell wall preparations. In this article, these proteins will be called CWPs. Although many CWPs (84) were found in both samples, only 36 and 17 were identified only in 5 and 11 day-old hypocotyls respectively.

**Table 1:** Number of proteins identified in cell wall fractions prepared from 5 and 11 day-old etiolated hypocotyls of *Arabidopsis*

	5 days	11 days	total
total number of identified proteins	147	126	173
number of predicted secreted proteins	120	101	137
number of predicted secreted proteins identified only in 5 or 11 day-old etiolated hypocotyls	36	17	

Proteins are listed in Additional data file 5.

**Figure 3:**
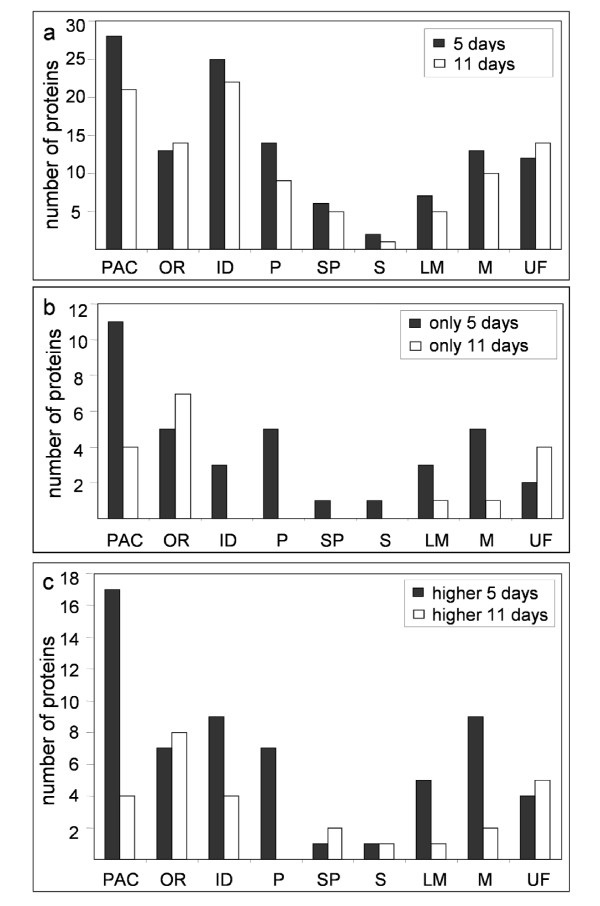
**Sorting of hypocotyl CWPs in functional classes.** Proteins were classified according to their functional domains as described in Experimental procedures: proteins acting on carbohydrates (PAC), oxido-reductases (OR), proteins with interaction domains (ID), proteases (P), structural proteins (SP), proteins involved in signaling (S), proteins related to lipid metabolism (LM), proteins with miscellaneous functions (M), and proteins of yet unknown function (UF). **a**. Number of proteins found in the nine functional classes in 5 (5 days), and in 11 day-old hypocotyls (11 days). **b**. Number of proteins found only in 5 (only 5 days), or in 11 day-old (only 11 days) hypocotyls. **c**. Number of proteins present in higher amount in 5 (higher 5 days) or in 11 day-old (higher 11 days) hypocotyls.

A second bioinformatics analysis of CWPs allowed their classification according to functional domains. For several protein families, experts' designation was used as described in Experimental procedures. The nine functional classes defined by Jamet *et al*. [12] were employed (Figure 3): proteins acting on carbohydrates, oxido-reductases, proteins with interaction domains, proteases, structural proteins, proteins involved in signaling, proteins related to lipid metabolism, proteins with miscellaneous functions, and proteins of unknown function. Some protein classes were more abundant at 5 days than at 11 days, *e.g*. proteins acting on carbohydrates (28 *vs *21), proteins with interaction domains (25 *vs *22), proteins related to lipid metabolism (7 *vs *5), and proteases (14 *vs *9) (Figure 3a). In each functional class, some CWPs were only identified at 5 or 11 days (Figure 3b). Differences appeared among proteins acting on carbohydrates (11 and 4 were found only at 5 or 11 days respectively), proteases (5 were only found at 5 days), proteins with domains of interactions with proteins or carbohydrates (3 were only found at 5 days), miscellaneous proteins and proteins of unknown function. In particular, the pattern of oxido-reductases appeared to be very different with 5 and 6 proteins being identified only at 5 and 11 days respectively. On the contrary, signaling and structural proteins showed minor changes. Due to their specific structural characteristics, they are not very present in either proteomes. Structural proteins are difficult to extract when they are cross-linked. They are also hard to identify because of numerous post-translational modifications (PTMs) [34]. Proteins involved in signaling, such as AGPs, may also have many PTMs [35], and proteins with trans-membrane domains are not usually extracted with the protocol used [14].

Finally, 51 proteins reported in this work were not identified in previous cell wall proteomic studies (Table 2). There are 11 CWPs that act on carbohydrates, 8 oxido-reductases, 5 proteases, 8 that carry interacting domains, 1 which is possibly involved in signaling, 1 structural protein, 4 proteins related to lipid metabolism, 5 proteins with diverse functions, and 7 without known function.

**Table 2:** CWPs identified in salt extracts of cell wall fractions prepared from 5 and 11 day-old etiolated hypocotyls of *Arabidopsis*

**accession**	**annotation**	**5 days**	**11 days**	**11 days *vs *5 days**
**AGI**				

**proteins acting on carbohydrates**				
At2g06850	GH family 16 (AtXTH4)	+	+	<
At5g13870	GH family 16 (AtXTH5)	+	-	
At3g44990	GH family 16 (AtXTH31)	+	-	
At2g36870	GH family 16 (AtXTH32)	-	+	
At1g10550	GH family 16 (AtXTH33)	+	+	<
At4g16260	GH family 17	-	+	
At4g19810	GH family 18 (chitinase)	+	-	
At3g12500	GH family 19 (chitinase)	+	+	≈
At3g55260	GH family 20 (beta-hexosaminidase)	+	-	
At1g19170	GH family 28 (polygalacturonase)	+	-	
At2g33160	GH family 28 (polygalacturonase)	+	-	
At3g06770	GH family 28 (polygalacturonase)	-	+	
At3g16850	GH family 28 (polygalacturonase)	+	+	≈
At3g61490	GH family 28 (polygalacturonase)	+	+	≈
At4g18180	GH family 28 (polygalacturonase)	+	-	
At1g68560	GH family 31 (alpha-xylosidase) (AtXYL1)	+	+	≈
At3g13790	GH family 32 (beta-fructofuranosidase)	+	+	≈
At5g34940	GH family 79 (endo beta-glucuronidase/heparanase)	+	+	<
At1g11580	CE family 8 (pectin methylesterase)	+	-	
**At1g53830**	CE family 8 (pectin methylesterase) (AtPME2)	+	+	<
At3g14310	CE family 8 (pectin methylesterase) (AtPME3)	+	+	≈
At3g43270	CE family 8 (pectin methylesterase)	+	+	≈
At4g33220	CE family 8 (pectin methylesterase)	+	+	≈
**At5g53370**	CE family 8 (pectin methylesterase)	+	-	
At4g37950	PL family 4 (rhamnogalacturonate lyase)	-	+	
At1g05570	GT family 48 (callose synthase) (AtCalS1)	+	+	≈
At5g02260	alpha-expansin (AtEXPA9)	+	+	<
At1g20190	alpha-expansin (AtEXPA11)	+	+	≈
At5g39270	alpha expansin (AtEXPA22)	+	-	
At3g45970	expansin-like A (AtEXLA1)	+	+	≈
At4g38400	expansin-like A (AtEXLA2)	+	+	<
At3g45960	expansin-like A (AtEXLA3)	+	-	
				
**oxido-reductases**				
At1g71695	peroxidase (AtPrx12)	+	+	>
At3g21770	peroxidase (AtPrx30)	+	+	≈
At3g32980	peroxidase (AtPrx32)	+	+	<
At3g49110	peroxidase (AtPrx33)	-	+	
At3g49120	peroxidase (AtPrx34)	+	+	≈
At3g50990	peroxidase (AtPrx36)	+	-	
At4g25980	peroxidase (AtPrx43)	-	+	
At4g30170	peroxidase (AtPrx45)	+	+	≈
At5g17820	peroxidase (AtPrx57)	+	-	
At5g64100	peroxidase (AtPrx69)	+	+	≈
At5g66390	peroxidase (AtPrx72)	-	+	
At2g30210	laccase homologue (AtLAC3)	+	+	≈
At1g30710	berberine-bridge enzyme homologue	+	-	
At4g20860	berberine-bridge enzyme homologue	-	+	
At5g44360	berberine-bridge enzyme homologue	+	+	>
At5g44410	berberine-bridge enzyme homologue	-	+	
At1g01980	berberine-bridge enzyme homologue	-	+	
At2g02850	plantacyanin ARPN (blue copper binding protein)	-	+	
At4g12880	early nodulin AtEN20 (blue copper binding protein)	+	+	<
At5g22140	expressed protein (oxido-reductase domain)	+	-	
At5g56490	expressed protein (FAD binding domain)	+	-	
				
**proteins with interacting domains**				
At1g53070	lectin homologue (legume lectin domains)	+	+	<
At1g78820	lectin homologue (curculin-like)	+	-	
At1g78830	lectin homologue (curculin-like)	+	+	≈
At1g78850	lectin homologue (curculin-like)	+	+	≈
At1g78860	lectin homologue (curculin-like)	+	+	<
At5g06860	PGIP1 (LRR domains)	+	+	≈
T23B7.10	PGIP1 homologue (LRR protein FLR1)	+	+	>
At5g12940	*Phaseolus vulgaris *PGIP2 homologue (LRR domains)	+	+	≈
At1g33590	expressed protein (LRR domains)	+	+	≈
At2g34930	expressed protein (LRR domains)	+	+	≈
At3g20820	expressed protein (LRR domains)	+	+	>
**At2g17120**	expressed protein (LysM domain)	+	+	<
At1g03220	carrot EDGP and tomato XEGIP homologue	+	+	≈
At1g03230	carrot EDGP and tomato XEGIP homologue	+	+	≈
At5g19110	carrot EDGP and tomato XEGIP homologue	+	+	<
At1g47710	serpin homologue (serine protease inhibitor)	+	+	<
At1g17860	inhibitor family I3 (Kunitz-P family)	+	+	≈
At1g73260	inhibitor family I3 (Kunitz-P family)	+	+	>
At1g47540	inhibitor family I18 (mustard trypsin inhibitor-2 family)	+	+	nd
At2g40880	inhibitor family I25 (phytostatin)	+	+	≈
At5g05110	inhibitor family I25 (phytostatin)	+	+	≈
At4g16500	inhibitor family I25 (cystatin family)	+	+	>
At4g25260	invertase/pectin methylesterase inhibitor homologue	+	+	<
At5g46940	invertase/pectin methylesterase inhibitor homologue	+	-	
At5g46960	invertase/pectin methylesterase inhibitor homologue	+	-	
				
**Proteases**				
At1g09750	aspartic protease homologue (pepsin family)	+	+	<
At3g02740	aspartic protease homologue (pepsin family)	+	-	
At3g52500	aspartic protease homologue (pepsin family)	+	-	
At3g54400	aspartic protease homologue (pepsin family)	+	+	≈
At5g07030	aspartic protease homologue (pepsin family)	+	+	≈
At1g79720	aspartic protease homologue (CND41 peptidase)	+	-	
At5g10770	aspartic protease (CND41 peptidase)	+	+	≈
At1g47128	cysteine proteinase homologue (papain family)	+	+	≈
At5g43060	cysteine proteinase homologue (papain family)	+	+	≈
At4g01610	cysteine proteinase homologue (papain family)	+	+	≈
At4g36880	cysteine proteinase homologue (papain family)	+	-	
At3g02110	serine carboxypeptidase D (SCPL25)	+	+	<
At5g23210	serine carboxypeptidase (SCPL34)	+	+	nd
At4g30610	carboxypeptidase homologue (BRS1 – Brassinosteroid-Insensitive BRI suppressor 1)	+	-	
				
**structural proteins**				
At1g28290	proline-rich protein	+	+	≈
At5g14920	proline-rich protein	+	+	≈
At2g05580	glycine-rich protein	+	+	>
At4g13340	LRR-extensin (AtLRX3)	+	-	
At3g24480	LRR-extensin (AtLRX4)	+	+	>
At4g18670	LRR-extensin (AtLRX5)	+	+	≈
				
**signaling**				
At4g05200	receptor kinase homologue (RLK, DUF26-1b subfamily)	+	-	
**At5g55730**	fasciclin-like arabinogalactan protein (AtFLA1)	+	+	>
				
**proteins related to lipid metabolism**				
At1g29670	lipase acylhydrolase homologue (GDSL family)	+	+	<
At1g54010	lipase/acylhydrolase homologue (GDSL family)	+	-	
At1g54030	lipase/acylhydrolase homologue (GDSL family)	+	+	≈
At3g48460	lipase/acylhydrolase homologue (GDSL family)	+	+	<
At5g15720	lipase/acylhydrolase homologue (GDSL family)	-	+	
At2g38530	non-specific lipid transfer protein type 1 (LTP2)	+	+	≈
At5g23820	expressed protein (ML domain – MD-2-related lipid recognition domain)	+	-	
At2g16001	expressed protein (lipid recognition domain)	+	-	
				
**miscellaneous functions**				
At2g27190	purple acid phosphatase homologue (PAP1)	+	+	<
At3g07130	purple acid phosphatase homologue	+	+	<
At5g34850	purple acid phosphatase homologue	+	-	
At4g29270	acid phosphatase homologue	+	+	<
At4g24340	phosphorylase homologue homologue	+	-	
At3g02870	myo-inositol monophosphatase homologue	+	-	
At5g09440	*Nicotiana tabacum *phi-I homologue	+	+	≈
At5g64260	*Nicotiana tabacum *phi-I homologue	+	+	≈
At5g66590	*Nicotiana tabacum *pathogenesis-related protein PR1 homologue	+	+	≈
At2g28790	*Lycopersicon esculentum *osmotin homologue	+	+	≈
At5g15230	gibberellin-regulated protein (GASA4)	+	-	
**At4g27110**	homologous to COBRA (AtCOBL10)	+	-	
At1g09560	germin (subfamily 2, member 1, GLP5)	-	+	
				
**unknown function**				
At3g56750	expressed protein	**-**	+	
At3g22000	expressed protein (DUF26)	**-**	+	
At1g26850	expressed protein (DUF248)	**-**	+	
At1g80240	expressed protein (DUF642)	+	+	nd
At3g08030	expressed protein (DUF642)	+	+	≈
At4g32460	expressed protein (DUF642)	+	+	<
At5g11420	expressed protein (DUF642)	+	+	≈
At5g25460	expressed protein (DUF642)	+	+	≈
At1g78460	expressed protein (SOUL heme binding domain)	-	+	
At2g04690	expressed protein (homologous to a human brain CREG protein)	+	+	≈
At2g15220	expressed protein (Plant Basic Secreted Protein domain)	+	+	>
At2g34700	expressed protein (Ole e1 allergen domain)	+	+	≈
At3g20370	expressed protein (MATH domain)	+	-	
At2g28490	expressed protein (cupin domain)	+	+	<
At3g22640	expressed protein (cupin domain)	+	+	≈
At4g36700	expressed protein (cupin domain)	+	-	

Accession numbers of genes encoding proteins predicted to have a GPI anchor or trans-membrane domains are in bold or in grey boxes respectively. Accession numbers of genes encoding proteins identified for the first time by using cell wall proteomics are underlined. Details of functional annotation are in Additional data files 1 and 2. + indicates the presence of proteins in 5 or 11 days-old hypocotyls. - means that the proteins were not identified. The relative amount of proteins in these two physiological stages is indicated in the right column (see Additional data file 6). nd means not determined.

### Semi-quantitative comparative analysis of CWPs

**Figure 4:**
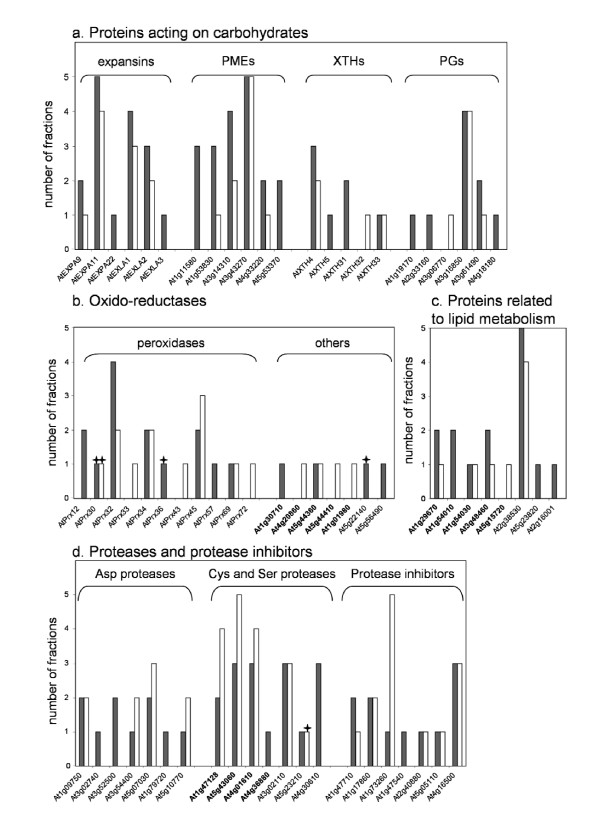
**Occurrence of CWPs of selected families of proteins extracted from hypocotyls. a**. Families of proteins predicted to encode expansins, PMEs, XTHs and PGs. **b**. Protein families predicted to encode oxido-reductases. Berberine-bridge enzymes are in bold. **c**. Protein families predicted to encode proteins related to lipid metabolism. Proteins having a GDSL Lipase/Acylhydrolase domain are in bold. **d**. Families of proteins predicted to encode proteases or protease inhibitors. Cys proteases are in bold. The number of FPLC fractions in which each protein was identified was counted: black and white bars respectively stand for 5 and 11 day-old etiolated hypocotyls. Stars indicate proteins that have only been identified after separation by 1D-E. Detailed information on the distribution of proteins in the total extract and in FPLC fractions are given in Additional data file 5.

In the previous section, the comparison of the two physiological stages was based on the single criterion of presence/absence of a protein among proteins identified by MS. A more precise comparison would require the quantification of the proteins. However, for several reasons, CBB staining of the gels does not allow such quantification. Despite improvement in the separation of proteins by liquid chromatography followed by 1D-E, as compared to 1D-E alone, most proteins were found in several FPLC fractions and in several bands of the same FPLC fractions. This was probably due to PTMs, proteolytic maturation or degradation of proteins. Moreover, due to differences in ionization efficiency of diverse peptides, and variations related to competitive desorption of peptides at the time of ionization, MALDI-TOF MS analyses are not quantitative. We propose alternative ways to compare the proteins of both samples. In a first approach, FPLC fractions, in which proteins were identified, were counted. This was done in order to give a first criterion, based on the following rationale: an abundant protein is more difficult to resolve and will be distributed in more fractions than a rare protein. The calculations carried out on members of several gene families give an overview of the relative abundance of each protein (Figure 4). Two additional criteria were then considered with the aim of evaluating the relative amount of each protein at both stages of development: the number of bands per fraction, and the percentage of coverage of the amino acid sequence by peptide mass mapping. A correlation was observed between the abundance of a protein and the number of matching peptides expressed as a percentage of coverage (results not shown). For a given protein, adding up all values, a semi-quantitative index (QI) was obtained, which allows comparisons of the relative amount of each protein in the two samples (Additional data file 6).

Taking into account QIs, 63% of the proteins showed differences in the two developmental stages: 61 proteins (42%) are more abundant at 5 days and 26 (17%) at 11 days. Figure 3c presents the results ordered by functional classes. These results were fully consistent with those described above, which only take into account the presence of a protein in FPLC fractions (Figure 3b). However, the differences are now more important. For example, 17 out of 32 for proteins acting on carbohydrates are more abundant at 5 than at 11 days. The case is the same for proteins having domains of interactions, proteins related to lipid metabolism, and miscellaneous proteins.

For proteins acting on carbohydrates (Figure 4a), all the identified expansins were more represented at 5 than at 11 days. The situation was similar for PMEs with the exception of At3g43270 that was found in the same number of FPLC fractions at 5 and 11 days. On the contrary, XTHs and PGs presented a more complex pattern. Three XTHs were more abundant at 5 than at 11 days (AtXTH4, AtXTH5, and AtXTH31), whereas AtXTH33 was equally present at both stages, and AtXTH32 was found only at 11 days. Three PGs were found only at 5 days (At2g33160, At1g19170, and At4g18180), At3g15850 was equally represented at 5 and 11 days, and At3g06770 was only found at 11 days.

The distribution of oxido-reductases in the two stages looked as complicated as that of XTHs (Figure 4b). AtPrx36 and AtPrx57 were identified only at 5 days whereas AtPrx12, AtPrx43, and AtPrx72 were identified only at 11 days. AtPrx32 was more abundant at 5 days whereas AtPrx45 was more abundant at 11 days. Finally, AtPrx30, AtPrx34, and AtPrx69 were equally present at both stages. Among other proteins predicted to be oxido-reductases, berberine-bridge enzymes were found in the two stages of development.

Most proteins related to lipid metabolism were more abundant at 5 than at 11 days (Figure 4c). This is especially the case for proteins containing a GDSL-Lipase/Acylhydrolase domain (At1g29670, At1g54010, and At3g48460).

In the case of proteases (Figure 4d), the situation differed depending on the protease family. Three Asp proteases were found only at 5 days (At1g79720, At3g02740, and At3g52500), whereas 3 of them were more abundant at 11 than at 5 days (At3g54400, At5g10770, and At5g07030). At1g09750 was equally present at both stages. Three Cys proteases were more abundant at 11 than at 5 days (At1g47128, At5g43060, and At4g01610). Instead, a Ser protease was found only at 11 days (At4g30610). Finally, protease inhibitors (Figure 4d) were distributed in the two stages of development, with 4 of them equally present at both stages, 2 only found at 5 days, and 1 more represented at 11 days.

## Discussion

This work provides a global picture of the cell wall proteome during elongation of etiolated hypocotyls of *Arabidopsis*. It shows the dynamics of CWPs during two phases of hypocotyl development, *i.e*. active elongation and after growth arrest. Expected CWPs known to be involved in cell wall extension such as XTHs, expansins, PGs, PMEs and peroxidases were identified as well as new CWPs such as proteases, proteins predicted to be related to lipid metabolism and proteins of unknown function. In addition, the occurrence of CWPs known to be related to cell wall extension after growth arrest showed that those proteins probably have other functions in mature cell walls.

Important progress in plant cell wall proteomics was achieved by setting up a new separation method for CWPs. Separation of plant CWPs for proteomic purposes was difficult using 2D-E [12]. The window of protein separation is optimal for pIs between 3 and 10 and for molecular masses between 120 and 10 kDa. Since most CWPs are basic glycoproteins, they tend to migrate as a smear on the basic side of 2D-gels [15]. Alternative methods were proposed; they consisted in separation of CWPs into an acidic and a basic fraction by cation exchange chromatography followed by 2D-E and 1D-E respectively [20,21]. The new method includes a first step of separation by cation exchange chromatography at acidic pH, and a second step of separation by 1D-E. It gives more information on the physico-chemical properties of the proteins, allows comparative semi-quantification among different samples, as well as a better identification through MALDI-TOF MS. In the case of etiolated hypocotyls of *Arabidopsis*, it allowed the doubling of the number of proteins identified as compared to separation by 1D-E alone. In addition, since many CWPs can now be visualized, this work provided preparative tools for developing biochemical studies on CWPs, either for further purification or structural characterization.

Altogether, 137 CWPs were identified in this study among which 51 had not been previously identified through cell wall proteomics. This work also presents an overview of the dynamics of CWPs during cell elongation. Many differences were observed between elongating and fully-grown hypocotyls. When only the presence/absence of a CWP was considered, these changes concerned 53 out of the 137 identified CWPs (38%). When the proposed semi-quantification method was taken into account, this percentage increased to 63% (34 additional proteins). Changes in the same gene family can reflect the regulation of gene expression at different stages of development and/or differences in biological activity, as discussed below for XTHs, PGs, expansins, PMEs, and peroxidases. Proteins acting on carbohydrates are more numerous and more abundant in elongating hypocotyls than in fully-grown hypocotyls. This was to be expected since rearrangements of cell wall polysaccharides are very important during cell elongation [7]. The fact that proteases are more numerous and in higher amounts at 5 than at 11 days is more surprising. Nothing is known about their targets in cell walls. Are they contributing to release peptides involved in signaling [36]? Are they involved in protein maturation [13] or in protein degradation? Conversely, two protease inhibitors are much more abundant at 11 than at 5 days. Altogether, it seems that proteolytic activities are more important when elongation is active than during elongation arrest. Among oxido-reductases, five berberine-bridge enzymes were identified among which three were present only at 11 days. The role of such proteins in cell walls is still unknown. For proteins with interacting domains, some lectins and PME inhibitors are more abundant at 5 days. Among miscellaneous proteins, the amount of CWPs containing phosphatase domains was found to be higher at 5 than at 11 days. Such proteins were shown to be associated to the regeneration of protoplast cell walls [37] and pollen tube growth [38], but their precise roles are still unknown. A protein homologous to COBRA (AtCOBL10) was only found at 5 days. Although the function of AtCOBL10 is not known, it should be noted that COBRA was shown to participate in the orientation of cellulose microfibrils, and dark-grown hypocotyls of the *cob-4 *mutant have a 95% reduction in length compared to the wild-type [39]. AtCOBL10 may play such a role during the elongation of hypocotyl cells.

Many proteins expected to participate in cell wall extension, such as XTHs, expansins, PGs, PMEs and peroxidases [3,8,9,40] were found. But such proteins, *i.e*. same proteins or proteins of the same family were also found after completion of elongation. Several hypotheses can be proposed. Although many proteases were identified at both stages of development suggesting a regulation of CWPs by proteolytic degradation, these proteins can have a long half-life. However, it is probable that some of these proteins participate in the differentiation of tracheary elements, such as AtXTH32 which was only identified at 11 days. This XTH which belongs to the phylogenic group 3, like AtXTH31 and AtXTH33, has been assumed to have xyloglucan endo-hydrolysis activity [41]. AtXTH31-33 might be involved in the rearrangement of cell walls of differentiating vessels elements. Such elements can be observed using microscopy (not shown). In the same way, some expansins were found in differentiating tracheary elements [42]. Finally, at least PMEs and peroxidases were assumed to play a role both during the elongation process and elongation arrest. The enzymatic activity of PMEs may be modulated, depending on the pH of the extracellular matrix and on the structure of pectic homogalacturonans. They could have either a local activity favoring the enzymatic activity of endo-PGs thus producing fragments of pectin, or a processing activity leading to the de-esterification of stretches of GalA and to the formation of the so-called egg-boxes that tend to rigidify the pectic network [8]. Moreover, the degree of pectin methyl-esterification was shown to be positively correlated to hypocotyl growth [43]. The activity of peroxidases is also versatile [9]. During the hydroxylic cycle, peroxidases can produce reactive oxygen species that can break cell wall polysaccharides in a non-enzymatic way thus favoring cell wall extension [9,44]. On the contrary, during the peroxidative cycle, peroxidases can promote cross-linking of cell wall components such as structural proteins or lignins. In addition, members of most of these protein families were identified in apoplastic fluids of rosette leaves [21]. Since leaf cells are surrounded by mature walls, this can mean that those CWPs may play house-keeping roles.

Proteomics provides information about possible regulatory mechanisms of CWPs. As previously discussed [10], the presence of a protein does not mean that it has full biological activity. Proteins with putative enzymatic activities are numerous, but inhibitors of these activities are also present. This is the case for proteases (14) and protease inhibitors (7), PMEs (6) and PME inhibitors (3 PMEIs). Some PMEs have a pro-domain consisting of a PMEI. However, such domains are assumed to be cleaved during or just after protein export, since they were never found in purified PMEs [8]. In the same way, no peptide matching the PMEI domains were found during identification of PMEs by peptide mass mapping (data not shown). Other enzyme inhibitors are assumed to be involved in defense reaction, such as PG inhibiting proteins (3 PGIPs) and inhibitors of xyloglycan endoglucanases (3 XEGIPs). Indeed, some of them were shown to be specifically active against fungal enzymes [45]. Other regulatory mechanisms include variations in pH of the extracellular matrix that occur during growth arrest [45], physical contact between enzymes and their substrates [10] and proteolytic degradation.

Eight proteins predicted to be related to lipid metabolism were identified at both stages of hypocotyl development. At present, little is known about the functions of such proteins in cell walls. Since etiolated hypocotyls have a thicker cuticle than light-grown hypocotyls [4], the presence of proteins involved in cuticle formation is expected. Several genes encoding proteins from the same families have been found to be up-regulated in 35S::*AtMYB41 *plants having defects in cell expansion and leaf surface permeability [46]. Two mutants affected in genes encoding proteins related to lipid metabolism have been described. *GLIP1 *encodes a predicted lipase/acylhydrolase that was shown to have a lipase activity *in vitro *and to disrupt fungal spore integrity at the level of cell wall and/or membrane [47]. Although none of the proteins of the GDSL family was shown to have an activity towards natural lipids *in vitro*, it cannot be excluded that such proteins are hydrolases acting on cutin or suberin lipids (F. Beisson, personal communication). *BODYGUARD *encodes a protein predicted to belong to an α/β-hydrolase fold superfamily [48]. The *bodyguard *mutant shows defects in cuticle formation that could result from incomplete polymerization of the carboxyl esters of the cuticle. The function of LTPs is still a matter of debate. They were shown to bind fatty acids and to transfer phospholipids among membranes *in vitro *[49]. *At2g38530 *encoding LTP2 was found to be up-regulated in the epidermis of stems and assumed to contribute to active cuticle formation during stem elongation [50]. Apart from this role in cuticle formation, many roles were proposed for LTPs including systemic resistance signaling [51], ability to promote cell wall expansion through binding to a hydrophobic partner in cell walls [52], and activation of a PG [53]. CWPs predicted to be related to lipid metabolism, identified in this study are candidates for roles in cuticle formation.

## Conclusion

This proteomic survey provides tools for biochemical studies of CWPs, identifies members of multigene families involved in cell elongation, and gives clues for unraveling the function of many CWPs in etiolated hypocotyls. It also shows CWP dynamics in the two developmental stages. This is illustrated by changes in protein amount, presence/absence of specific members of multigene families, and presence of many enzymes including proteases and their inhibitors. Interestingly, many CWPs were found only at one stage of development, either in active elongation or after growth arrest. Conversely, different CWPs from the same gene families were found at both stages of development, showing stage-specific regulation and suggesting diverse roles in cell walls. Particular attention should be paid to proteins of unknown function, some of which are very abundant. Additional functional studies are now required to shed light on the roles of the identified CWPs during the elongation of hypocotyls in the dark.

## Abbreviations

AGP: arabinogalactan protein; CBB: Coomassie brilliant blue; CWP: cell wall protein; 1D-E: mono-dimensional electrophoresis; 2D-E: two-dimensional electrophoresis; FLA: fasciclin arabinogalactan protein; GH: glycoside hydrolase; GRP: glycine-rich protein; LAE: late-abundant embryogenesis protein; LTP: lipid transfer protein; LRR: leucine-rich repeat; LRX: leucine-rich repeat extensin; MALDI-TOF: matrix-assisted laser desorption ionization – time of flight; PG: polygalacturonase; PL: polysaccharide lyase; PME: pectin methylesterase; PTM: post-translational modification; XTH: xyloglucan endotransglucosylase-hydrolase.

## Authors' contributions

MI performed cell wall preparations, protein extraction and separation, as well as preparation of samples for MS. HC analyzed all the MALDI-TOF MS spectra. GB carried out protein separation by high performance chromatography and provided help in sample preparation for MS. RP-L contributed to data interpretation and to writing the manuscript. EJ performed the bioinformatics analyses and drafted the manuscript. All authors read and approved the final manuscript.

## Supplementary Material

Additional file 1 Identification of proteins extracted from the cell wall fraction prepared from 5 day-old Arabidopsis hypocotyls by CaCl_2 _and LiCl solutions. Click here for file

Additional file 2 Identification of proteins extracted from the cell wall fraction prepared from 11 day-old Arabidopsis hypocotyls by CaCl_2 _and LiCl solutions. Click here for file

Additional file 3 Identification of proteins extracted by CaCl_2 _and LiCl from the cell wall fraction prepared from 5 day-old *Arabidopsis *etiolated hypocotyls by MALDI-TOF MS. Click here for file

Additional file 4 Identification of proteins extracted by CaCl_2 _and LiCl from the cell wall fraction prepared from 11 day-old *Arabidopsis *etiolated hypocotyls by MALDI-TOF MS. Click here for file

Additional file 5 Classification of proteins extracted from cell wall fractions prepared from 5 and 11 day-old Arabidopsis hypocotyls in FPLC fractions A to Z. Click here for file

Additional file 6 Semi-quantification of CWPs identified by MALDI-TOF MS from 5 and 11 day-old etiolated Arabidopsis hypocotyls. Click here for file
