# Supplementary material for: A new picture of cell wall protein dynamics in elongating cells of Arabidopsis thaliana: Confirmed actors and newcomers
Source: BMC Plant Biol. 2008 Sep 16;8:94. doi: 10.1186/1471-2229-8-94 (PMC2551616; doi:10.1186/1471-2229-8-94)
Supplement: Additional file 4 — Identification of proteins extracted by CaCl2 and LiCl from the cell wall fraction prepared from 11 day-old Arabidopsis etiolated hypocotyls by MALDI-TOF MS. [file 1471-2229-8-94-S4.pdf]

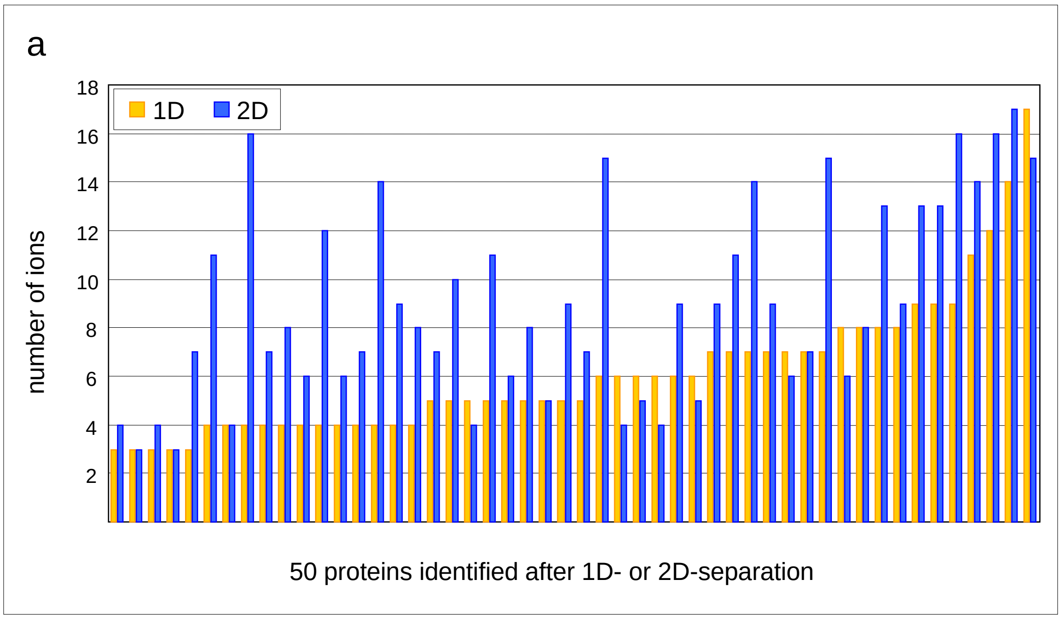

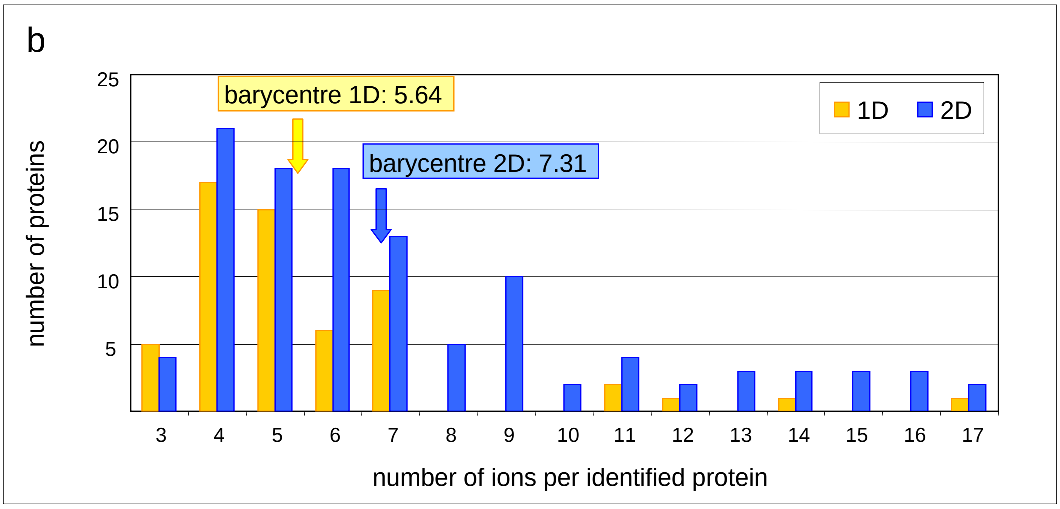

Additional data file 4

Identification of proteins extracted by $CaCl_2$ and LiCl from the cell wall fraction prepared from 11-day-old etiolated hypocotyls by MALDI-TOF MS. a. Improvement of efficiency of protein identification after 2D-separation of proteins. Numbers of ions found for identification of proteins after 1D- (yellow bars) and 2D- (blue bars) separation are shown. Note that only 7 out of 50 proteins are identified with less ions after 1D- than after 2D-separation. **b.** Increase in number of ions for protein identification after 2D-separation of proteins. Number of proteins identified with 3 to 20 ions are shown after 1D-(yellow bars) and 2D- (blue bars) separation. Note that the barycentre of this number of ions used for successful identification is higher after 2D- than after 1D-separation.
